# Case Report: Mechanisms in Misdiagnosis of Autism as Borderline Personality Disorder

**DOI:** 10.3389/fpsyg.2022.735205

**Published:** 2022-02-04

**Authors:** Stine Iversen, Arvid Nikolai Kildahl

**Affiliations:** ^1^Regional Section Mental Health, Intellectual Disabilities/Autism, Oslo University Hospital, Oslo, Norway; ^2^NevSom Norwegian Centre of Expertise for Neurodevelopmental Disorders and Hypersomnias, Oslo University Hospital, Oslo, Norway

**Keywords:** autism, personality disorder, assessment, mental health, self-injurious behavior, misdiagnosis

## Abstract

Autistic individuals without intellectual disabilities are sometimes not diagnosed until adolescence/adulthood. Due to increased risk of co-occurring mental health problems, these individuals may initially be referred to general, mental health services and not always be identified as autistic; some may be misdiagnosed with personality disorder (PD) prior to identification of autism. To explore possible mechanisms in misdiagnosis of autism, we report on the case of a young man with severe, non-suicidal self-injury (NSSI) and attention deficit disorder (ADD) who had been diagnosed with and treated for borderline PD prior to being diagnosed with autism. Following reassessment by mental health clinicians with experience of working with autistic individuals, the patient was diagnosed with autism, ADD, and depression—but not PD. Experiences from this case suggest that presence of co-occurring NSSI, depression, and ADD, as well as lack of comprehensive assessment and lack of autism knowledge in general mental health services, may contribute to risk that autism is misdiagnosed as PD. These findings highlight the need for autism expertise in general mental health services to facilitate appropriate diagnosis for autistic individuals who encounter these services, as well as the importance of undertaking comprehensive assessments.

## Introduction

Outcomes applicable to real-world contexts, with the potential of contributing to improvement in the lives of autistic individuals, are viewed as increasingly important in autism research (Roche et al., 2021). Also, there is a growing recognition that collaboration with the autistic community is an important part of the research process. Access to and expertise within services, including how individuals’ needs can be met in these services, have been found to be important areas of research for the autism community (Pellicano et al., 2014). A recent review identified the mental health of autistic people, as well as accurate identification and knowledge of autism, as important priorities (Roche et al., 2021).

It is not uncommon for autistic individuals without co-occurring intellectual disabilities to be diagnosed in adolescence/adulthood (Huang et al., 2020). As autism is associated with increased prevalence of psychiatric disorder (Lever and Geurts, 2016; Rosen et al., 2018), many may first be referred to mental health services due to these co-occurring difficulties rather than because of their autism (Huang et al., 2020; see also Stagg and Belcher, 2019; Henley, 2020; Tromans and Chester, 2020). However, autism characteristics may be confused with symptoms of psychiatric disorder by mental health professionals (Helverschou et al., 2011; Au-Yeung et al., 2019), and autism may thus not always be recognized (Fusar-Poli et al., 2020). Assessment and understanding of the specific individual’s autism characteristics are likely to be a prerequisite for differentiation between these characteristics and mental health symptoms, and thus also for adequate diagnosis of co-occurring mental health problems in these individuals (Helverschou et al., 2011; Rosen et al., 2018).

Differentiating autism and psychiatric disorder may be challenging (Helverschou et al., 2011; Rosen et al., 2018) and may be particularly challenging for personality disorders (PDs; Lugnegård et al., 2012; Da Cagna et al., 2019; Gordon et al., 2020). The relationship between autism and PD is poorly understood, and recent studies have led to growing awareness that they share surface symptom similarities contributing to challenges in differential diagnostic assessment (Rydén et al., 2008; Lugnegård et al., 2012; Da Cagna et al., 2019; Gordon et al., 2020). Both conditions affect the ways in which the individual communicates and interacts with other people (American Psychiatric Association, 2013; Da Cagna et al., 2019) and thus may impact several aspects of life, including friendships, intimate relations, and work.

Furthermore, autism and PD both refer to stable characteristics (American Psychiatric Association, 2013; Da Cagna et al., 2019), which makes approaches typically applied to differentiate autism and psychiatric disorder less helpful, i.e., where the clinician looks for changes to level of functioning, behavior, or autism symptomatology (Helverschou et al., 2011). However, autism characteristics are usually to some degree present and observable from early childhood, while symptoms of PD typically manifest during adolescence (Da Cagna et al., 2019; Gordon et al., 2020). A detailed developmental history may therefore be helpful to distinguish these conditions (Da Cagna et al., 2019). In adults, however, this may be challenging because retrospective reports from caregivers alone may not be sufficiently reliable or informative when it comes to early development (Fusar-Poli et al., 2017).

Recent findings indicate that PD may be a relatively common misdiagnosis in autistic adults before their autism is recognized (Kentrou et al., 2021). The current case concerns a young man who had been diagnosed with and treated for borderline PD for several years, before he was reassessed and diagnosed with autism. The current study aims to explore the possible mechanisms in the previous misdiagnosis, to identify potentially contributing mechanisms.

The study was approved by the Data Protection Official at the Oslo University Hospital (#20/14349). The patient has been anonymized.

## Case Description

At referral, “Adrian” was in his early 20s. In childhood, he displayed delayed language development and concentration problems, and was diagnosed with attention deficit disorder (ADD), dyslexia, and specific learning difficulties. Adrian described these difficulties as resulting in low self-esteem: “I could not read or write like the other kids in school. I felt stupid.” During adolescence, he developed symptoms of anxiety and depression and started displaying non-suicidal self-injury (NSSI). This resulted in several admittances to acute psychiatric inpatient wards. Adrian also had a history of suicide attempts. At 18, he was diagnosed with borderline PD. During a later admission, Adrian completed the Ritvo Autism Asperger Diagnostic Scale-Revised (RAADS-R; Ritvo et al., 2011) but scored below the cutoff value for autism. No further autism assessment was undertaken. Assessment of Adrian’s intellectual abilities indicated functioning in the low average area.

Adrian had been treated with various kinds of antidepressants, antipsychotics, and anxiolytics. In addition to frequent hospitalizations, he had received outpatient therapy for borderline PD. Adrian lived alone and had daily visits from municipal mental health services which mainly involved delivery of medication. He had a girlfriend and two friends he occasionally spent time with, but described difficulties initiating contact. A lot of Adrian’s time was spent engaging in NSSI (typically cutting himself) and seeking out emergency medical and mental health services. Episodes involving NSSI would occur up to 14 times a month, sometimes with more episodes in 1 day if the first episode did not result in a certain number of stitches.

Adrian described his previous contacts with the mental healthcare system as often leading to feelings of rejection. He frequently experienced not being heard or listened to, with mental health professionals emphasizing their own understanding of his difficulties rather than exploring Adrian’s own views. This included a primary focus on risk and management of NSSI, while paying less attention to other difficulties.

The current, inpatient assessment in a specialized ward included interviews with Adrian, his family and professional caregivers, use of structured assessment tools, and direct observation by clinicians. The team included a psychologist, a psychiatrist, psychiatric nurses, social education nurses, and experienced nursing assistants. Assessment tools included autism diagnostic tools, conventional assessment tools for mental disorders, and one tool developed for assessment of mental disorders in autistic people.

Adrian described feeling lonely as an important trigger for NSSI. He never communicated to others about it prior to engaging in NSSI, and NSSI most often occurred when he was alone in the evenings or at night. NSSI was not reported to occur as a response to interpersonal conflict. During interviews, it became evident that Adrian had difficulties recognizing and discerning emotions. He had few words for his inner states and expressed that emotions were difficult to manage: “even joy is difficult, emptiness is better.” Adrian described fear of being a burden in friendships and relationships with caregivers, and there often was a discrepancy between his inner states and what he communicated to others. In general, Adrian rarely experienced conflicts in his interactions with other people but described reacting to interpersonal difficulties by withdrawing and letting the relationship “fade out.”

The autism diagnostic tools included the Autism Diagnostic Observation Schedule-2 (ADOS-2; Lord et al., 2012), a semi-structured interaction observation and interview, and the Autism Diagnostic Interview-Revised (ADI-R; Lord et al., 1994), a semi-structured interview with caregivers. The ADOS-2 was done with Adrian himself, while the ADI-R was completed with his parents. In addition, a detailed developmental history was obtained from Adrian’s parents, Adrian himself, and existing medical records and previous assessments. Adrian’s scores on the ADOS-2 and the ADI-R indicated presence of a potential autism spectrum disorder, see Table 1. While the diagnostic algorithm of the ADI-R focuses on the ages 4–5, Adrian’s parents described that several of the relevant behaviors became more evident in Adrian’s adolescence, making the “current” score for the ADI-R higher than the one used in the diagnostic algorithm. The clinician completing the ADOS-2 remarked that while Adrian seemed to display several appropriate strategies for social interaction, he tended to use similar strategies throughout the assessment situation, even when they could be perceived by others as less appropriate. Direct observation in the ward indicated that Adrian displayed several good strategies for coping in social interaction but had more extensive difficulties with communication and flexibility. Observations in the ward and during individual therapy also indicated that Adrian’s mentalization abilities (i.e., his abilities to make inferences regarding his own and others’ mental states; Gordon et al., 2020) did not fluctuate with emotional states or level of emotional activation. Thus, these observations were in line with the results from the ADI-R and the ADOS-2, suggesting that Adrian had difficulties in social interaction and communication, but that his difficulties in communication were more extensive than his difficulties in other aspects of social interaction.

**Table 1:** Scores on the Autism Diagnostic Observation Schedule-2 (ADOS-2) and Autism Diagnostic Interview-Revised (ADI-R), with cutoff values.

Scores and cutoffs on the ADOS-2 and ADI-R subscales			
	Score	Cutoff autism	Cutoff autism spectrum
Autism Diagnostic Observation Schedule-2			
Communication	5	3	2
Reciprocal social interaction	4	6	4
Communication + Reciprocal social interaction	9	10	7
Creativity	0	N/A	N/A
Restricted and repetitive behaviors	0	N/A	N/A
Autism Diagnostic Interview-Revised			
Reciprocal social interaction	6	10	
Communication	8	8	
Restricted, repetitive and stereotyped behaviors	3	3	
Atypical development apparent at or before 36 months	2	1	

*The ADOS-2 uses two different cutoff values, while the diagnostic algorithm of the ADI-R only uses one. Module 4 of the ADOS-2, for adolescents and adults with fluent verbal language, was used*.

For assessment of PD, the Structured Clinical Interview for DSM-IV Axis II Personality Disorders (First and Gibbon, 2004) was used as an interview with Adrian himself. Adrian reported symptoms in various domains, including schizoid, avoidant, dependent, and borderline PD. However, he did not report sufficient symptoms to meet criteria for any of these disorders. On the Mini International Neuropsychiatric Interview (Sheehan et al., 1998), Adrian reported symptoms of anxiety, depression, and dysthymia. No traumatic experience according to the diagnostic criteria for post-traumatic stress disorder was reported, but Adrian described lifelong difficulties with feeling stupid, out of place, and having difficulties interacting with other people. On the Montgomery and Åsberg Depression Rating Scale (Montgomery and Åsberg, 1979), Adrian scored 24, indicating moderate depression. Further assessment tools included an autism-specific screening tool for mental disorder, the Psychopathology in Autism Checklist (Helverschou et al., 2009), and assessment tools for trauma-related disorders. These yielded no further information relevant to the diagnostic formulation.

## Timeline

**Table:** 

Age	Incident/diagnoses
3	Delayed language development.
10	Dyslexia diagnosed. Outpatient treatment for emotional problems.
11	Attention deficit disorder (ADD) diagnosed.
16	Debut of non-suicidal self-injury (NSSI).
17	Debut of what was later understood as recurrent depressive disorder. First suicidal attempt. First admittance to an acute psychiatric ward.
18	Increasing frequency and severity of NSSI. Diagnosed with borderline personality disorder (BPD).
20	Autism suspected. Negative screening.
22	Referred for specialized assessment. Autism diagnosed, with co-occurring ADD and depressive disorder. BPD removed as diagnosis.

## Diagnostic Formulation and Treatment Plan

It was concluded that Adrian met criteria for an autism spectrum disorder, co-occurring ADD, and depressive disorder but not borderline PD. Prior to referral, Adrian had been prescribed olanzapine 10 mg for agitation and restlessness, fluoxetine 40 mg for depressive symptoms, quetiapine 200 mg for sleep, and levomepromazine 50 mg for sleep. Use of a wide range of other psychopharmacological medications had previously been attempted. Because Adrian had possible side effects (lowered energy and slurred speech) and because he was not assessed to have a psychotic disorder, treatment with antipsychotics (olanzapine and quetiapine) was discontinued. Within a few weeks, a slight change in Adrian’s demeanor was observed; he took more social initiatives and seemed more articulate. No negative effects of discontinuation were observed. According to both himself and informants, fluoxetine treatment seemed to have some effect on Adrian’s depressive symptoms and was therefore continued, as was levomepromazine to help him sleep.

Individually adapted mental health nursing strategies emphasized providing structure, aiding Adrian in regulating his emotions and performing enjoyable activities. This included maintaining a low degree of criticism and demands, making suggestions of activities while putting little pressure on Adrian. Physical activity, regular meals, and maintaining good sleep habits were emphasized. Adrian agreed to practice making contact with nursing staff whenever he felt anxious or had an impulse to engage in NSSI. In these instances, staff focused on helping Adrian to identify and experience alternative strategies for relieving inner tension, an approach inspired by strategies from dialectical behavior therapy (Iversen et al., 2019; see also Hartmann et al., 2012; Bemmouna et al., 2021).

Adrian had sessions with a therapist 2–3 times a week during the stay, focusing on psychoeducation about autism and depression, as well as identification, recognition, and regulation of emotions. The latter was achieved by Adrian and the therapist making an individually adapted booklet with overview of the different emotions, their functions, how Adrian experienced them, and possible strategies to manage them. The individual therapist and nursing staff collaborated closely throughout the admission, for instance by helping Adrian test potential emotion regulation strategies identified during therapy in other settings. Adrian cut himself only once during the stay. This episode occurred during one of the times he was on leave from the ward.

## Discussion

The current patient, a young man with severe NSSI who previously had received a diagnosis of borderline PD, was assessed to meet criteria for autism and depressive disorder. This changed understanding and adaptation of treatment seemed to have positive consequences for the frequency of NSSI, as well as for his relationships with family and caregivers, and led to a change in his strategies for seeking assistance. Lack of previous, comprehensive assessment and the patient displaying severe NSSI, in part possibly due to co-occurring depressive disorder and ADD, seemed to have contributed to misdiagnosis of autism as borderline PD.

In the current assessment, the combination of standardized assessment tools for autism and PD, going through the patient’s history, clinical observation, and use of informants together proved sufficient to differentiate autism and borderline PD. Obtaining a thorough developmental history using multiple sources was important, as was use of the ADOS-2 (see Fusar-Poli et al., 2017; Da Cagna et al., 2019). However, the assessment was carried out by a team with specific expertise and experience in mental health problems in autistic individuals, indicating a need for this expertise also in general mental health services. Experiences from this assessment suggest that it may be necessary to collect comprehensive information regarding signs and symptoms of both conditions in order to differentiate them or determine whether they co-occur (Da Cagna et al., 2019; Gordon et al., 2020): The current patient’s mentalization abilities did not seem to fluctuate with his emotional state, and he displayed reduced sharing of emotions rather than intense emotional involvement. However, during admission to acute mental health services prior to the current assessment, the patient had frequently been in a state of crisis, likely obscuring these nuances from the professionals treating him. Thus, it was necessary to observe and interact with the patient in an environment where he felt safe and taken care of to distinguish autism and PD.

Moreover, the patient’s difficulties with regard to social engagement were pervasive rather than transient; he did not display a pattern of attachment and rejection in interpersonal relationships, and his NSSI rarely occurred as a response to interpersonal conflicts. While his NSSI did seem to serve a function in regulation of emotion, this rarely involved others and he would often hide occurrences of NSSI from significant others (see Gordon et al., 2020). Finally, borderline PD is described to be associated with chronic feelings of emptiness (American Psychiatric Association, 2013). The current patient did not report problems with feelings of emptiness but seemed to pursue such feelings to avoid other, problematic emotions.

No clear, distinctive features have been identified to easily differentiate NSSI in autism and borderline PD, and the mechanisms in development of NSSI seem to share significant overlap between these respective conditions (Wilcox et al., 2012; Moseley et al., 2019). The current case indicates that presence of NSSI may be one factor contributing to risk that autism is misdiagnosed as borderline PD. NSSI in autistic individuals seems to be associated with impulsivity and lowered mood (Licence et al., 2020), suggesting that individuals with co-occurring depression and/or ADD/ADHD may be particularly at risk for this misdiagnosis. While it is unclear whether later autism diagnoses are associated with an increased risk of NSSI (Moseley et al., 2019; Licence et al., 2020; Hosozawa et al., 2021), the mechanisms described by the current patient as contributing to development of NSSI suggest that undiagnosed autism may have played a significant part. Thus, the current case indicates that further exploration of associations between later autism diagnoses and risk of NSSI is warranted, including whether there may be a subset of individuals with difficulties involving impulsivity and depression particularly at risk.

Undiagnosed autistic individuals displaying NSSI may initially be referred to general mental health services, and the current case highlights the need for professionals in these services to have the knowledge necessary to recognize and diagnose autism (see also Takara et al., 2015; Stagg and Belcher, 2019). The patient received treatment for borderline PD without any observable improvement in his difficulties for several years. In line with previous suggestions (Rydén et al., 2008; Fusar-Poli et al., 2020), screening for autism may thus be warranted in patients with NSSI and assumed PD not benefitting from attempted treatment. However, as in the current case, screening using only a single instrument and not conducting a comprehensive, differential diagnostic assessment may be insufficient (see also Fusar-Poli et al., 2017; Da Cagna et al., 2019).

Finally, the current patient reported experiencing mental health professionals as being primarily focused on risk management, and often feeling that he was not listened to. While risk management is important in instances of severe NSSI, listening to the views of people with NSSI is also important for understanding of triggers for NSSI. Thus, failure to listen to the patient on the part of mental health professionals may constitute another potential mechanism in misdiagnosis of autism as PD.

### Limitations and Strengths

The current study concerns a single case and therefore has limited generalizability, but its findings may be transferrable on a case-to-case basis to clinicians undertaking similar assessments (Maxwell and Chmiel, 2014). The exploration of a single case provided further insight into some of the possible mechanisms of misdiagnosis in autistic adults described by previous literature (e.g., Fusar-Poli et al., 2020).

### Conclusion

This case highlights the importance of autism knowledge in general psychiatric services. These services may be the first to encounter young, autistic adults who are yet to receive the appropriate diagnosis and have developed co-occurring difficulties, such as depression or NSSI. The ability of clinicians in these services to recognize signs of autism may thus be vital to these patients’ course of treatment and later outcomes. This case further underlines the importance of conducting comprehensive assessments when autism is suspected and provides an example that NSSI may constitute a severe and potentially lethal problem for undiagnosed autistic individuals, as well as how the appropriate diagnosis and understanding of these individuals’ difficulties may contribute to alleviating this problem. Finally, experiences from this case suggest that possible mechanisms in misdiagnosis of autism as PD may include lack of comprehensive assessment, lack of autism knowledge in general mental health services, and misinterpretation of commonly co-occurring conditions in autism, such as NSSI, ADD, and depression. Contact with mental health professionals primarily in acute phases involving NSSI, when the patient had frequently been in a state of crisis, may also have contributed to this misinterpretation of his difficulties, as may a lack of exploration of the patient’s views and his own understanding of his behaviors.

## Patient Perspective

Both Adrian and his parents reported finding the autism diagnosis helpful, as well as being a more appropriate description of how they understood his difficulties. The authors collaborated with Adrian in the writing of this manuscript, discussing the possibility of writing it before he provided written consent. Adrian read and provided important feedback on the manuscript, in particular the sections about how he experienced previous contacts with mental health professionals. Adrian approved the final version of the manuscript prior to submission.

## Data Availability Statement

The original contributions presented in the study are included in the article/supplementary material, and further inquiries can be directed to the corresponding author.

## Ethics Statement

The study, involving human participants, was reviewed and approved by the Data Protection Official, Oslo University Hospital. The patient/participant provided their written informed consent to participate in this study. Written informed consent was obtained from the individual for the publication of any potentially identifiable images or data included in this article. Written informed consent was obtained from the participant for the publication of this case report.

## Author Contributions

SI and AK jointly planned the current study and conducted the analysis. SI prepared the first draft of the manuscript, while SI and AK jointly revised and finalised it. All authors contributed to the article and approved the submitted version.

## Funding

The current study was funded by the authors’ employers through their work as clinicians. Open access publishing was funded by the Oslo University Hospital.

## Conflict of Interest

The authors declare that the research was conducted in the absence of any commercial or financial relationships that could be construed as a potential conflict of interest.

## Publisher’s Note

All claims expressed in this article are solely those of the authors and do not necessarily represent those of their affiliated organizations, or those of the publisher, the editors and the reviewers. Any product that may be evaluated in this article, or claim that may be made by its manufacturer, is not guaranteed or endorsed by the publisher.
